# AdpA, key regulator for morphological differentiation regulates bacterial chromosome replication

**DOI:** 10.1098/rsob.120097

**Published:** 2012-07

**Authors:** Marcin Wolański, Dagmara Jakimowicz, Jolanta Zakrzewska-Czerwińska

**Affiliations:** 1 Ludwik Hirszfeld Institute of Immunology and Experimental Therapy, Polish Academy of Sciences, ul. Weigla 12, 53114 Wrocław, Poland; 2 Faculty of Biotechnology, University of Wrocław, ul. Tamka 2, 50137 Wrocław, Poland

**Keywords:** AdpA, differentiation, *Streptomyces*, regulation, initiation of chromosome replication

## Abstract

AdpA, one of the most pleiotropic transcription regulators in bacteria, controls expression of several dozen genes during *Streptomyces* differentiation. Here, we report a novel function for the AdpA protein: inhibitor of chromosome replication at the initiation stage. AdpA specifically recognizes the 5′ region of the *Streptomyces coelicolor* replication origin (*oriC*). Our *in vitro* results show that binding of AdpA protein decreased access of initiator protein (DnaA) to the *oriC* region*.* We also found that mutation of AdpA-binding sequences increased the accessibility of *oriC* to DnaA, which led to more frequent replication and acceleration of *Streptomyces* differentiation (at the stage of aerial hyphae formation). Moreover, we also provide evidence that AdpA and DnaA proteins compete for *oriC* binding in an ATP-dependent manner, with low ATP levels causing preferential binding of AdpA, and high ATP levels causing dissociation of AdpA and association of DnaA. This would be consistent with a role for ATP levels in determining when aerial hyphae emerge.

## 2. Introduction

Transmission of genetic material to nascent cells requires precise regulation of chromosome replication and its coordination with the cell cycle. Any disturbance in these processes may lead to aneuploidy, or even to cell death caused by the lack of genetic material. Chromosome replication in all three domains of life is mainly regulated at the initiation step.

In bacteria, chromosome replication is initiated by cooperative binding of the initiator protein, DnaA, to multiple sequences termed DnaA boxes—9-mers within the *oriC* (origin of chromosomal replication) region. DnaA activity, like that of other initiator proteins, is regulated by binding and hydrolysis of ATP [1]. Only ATP-bound DnaA is able to unwind the duplex DNA within the *oriC* region, whereas the DnaA-ADP form is inactive [2,3]. ATP-binding allows the DnaA transition from a monomeric state to a large oligomeric complex that remodels replication origins and triggers duplex melting assembly [4]. Several factors that regulate replication initiation by binding directly to DnaA or *oriC* have been identified [5]. Among bacteria, the regulation of initiation of chromosome replication is best understood in *Escherichia coli* (for review, see [6]). Some *E. coli* regulatory systems, such as inactivation of DnaA–ATP by ATP hydrolysis, presumably exist in other bacteria (for review, see [7]). However, in bacteria that undergo a complex life cycle, the replication initiation regulatory network is likely to be more intricate than that in *E. coli*. These organisms require specific mechanisms that adjust the frequency of initiation of chromosome replication to developmental changes. *Bacillus subtilis* is one noteworthy example of such an organism. In this bacterium, the formation of endospores must be preceded by completion of the fifinal round of replication with concurrent prevention of a new round of replication at the initiation step. Two proteins, Spo0A [8] and SirA [9], involved in *B. subtilis* sporulation inhibit chromosome replication at the initiation step by binding to the *oriC* and DnaA, respectively. Interestingly, it has been shown that the *B. subtilis* Soj (ParA orthologue), required for accurate chromosome segregation, regulates DnaA activity [10]. Recently, Scholefield *et al*. [11] demonstrated that a direct interaction of Soj with the ATP-binding domain of DnaA stalls initiation of replication by inhibiting formation of DnaA oligomeric complex.

An intriguing case is provided by the complex cycle of morphological differentiation undergone by *Streptomyces*. These bacteria form a sporulating mycelium resembling that of fifilamentous fungi, raising questions about the mechanisms that coordinate the basic cellular processes of replication, segregation, cell division and the developmental programme. Streptomycetes grow by tip extension and hyphal branching to form vegetative hyphae in which septation occasionally occurs. Such growth-associated septation separates adjacent compartments containing several copies of the large (8–9 Mbp), linear chromosome into apical and non-apical compartments. In young mycelium, subapical compartments usually continue growth by the emergence of a new tip as a branch. In older parts of the substrate mycelium, branch emergence may not follow compartment subdivision immediately, and replication in such ‘blind’ compartments therefore needs to be delayed as well. Upon nutrient depletion, the surfaces of *Streptomyces* colonies differentiate to form aerial hyphae, which are subsequently converted into chains of spores. The aerial hyphae probably emerge from blind compartments, and their rapid extension is accompanied by intensive chromosome replication [12]. A single aerial tip compartment may contain 50 or more uncondensed, non-segregated chromosomes. After an aerial hypha has stopped growing, dozens of chromosomes are condensed and uniformly distributed along the hyphal tip prior to sporulation septation, ensuring that each pre-spore compartment receives a single chromosome. Conversion of multigenomic aerial hyphae into chains of unigenomic spores requires the inhibition of new rounds of replication, presumably at the initiation step. Thus, there are at least two developmental stages at which replication needs to be prevented. To date, however, our understanding of the mechanisms involved in coordinating the regulation of replication with growth and differentiation in *Streptomyces* has been very limited.

The transition from vegetative to aerial growth is initiated by a signalling cascade that depends on *bld* genes; these genes were so called because mutations in a variety of *bld* genes result in colonies with a ‘bald’ phenotype [13,14]. Among these genes, *adpA* (previously known as *bldH*) plays a key coordinating role in the regulation of *Streptomyces* morphological differentiation. AdpA, which was initially identified in *Streptomyces griseus* as a pleiotropic transcription factor [15,16], belongs to the AraC/XylS family of transcription regulators, whose members contain a dual helix–turn–helix motif in the C-terminal DNA-binding domain. In both *S. coelicolor* and *S. griseus*, AdpA is a master transcription factor that controls dozen of genes [17], whose products are required for morphological development, such as proteases and protease inhibitors (*sti1* [18], *clpP1* [19], *sgmA* [20]). In *S. griseus*, the AdpA additionally controls genes involved in secondary metabolite synthesis, including antibiotic production (e.g. streptomycin) [21,22].

**Figure 1.**
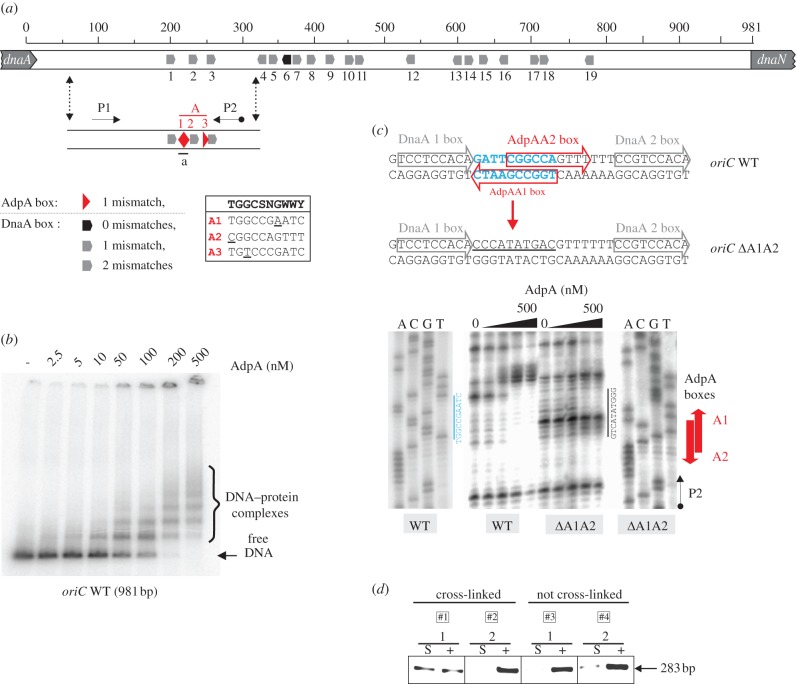
AdpA interacts specifically with the *oriC* region *in vivo* and *in vitro*. (*a*) Schematic of the *S. coelicolor oriC* region. The positions of AdpA and DnaA protein-binding sites (boxes) are presented by red triangles and black/grey pentagons (spatial arrangement and numbering of DnaA boxes adapted from Zawilak-Pawlik *et al*. [23]), respectively. In the rectangle next to legend, AdpA consensus sequence [22] (bold and upper one) and A1–3 boxes were aligned; underlined nucleotides indicate mismatches. Radiolabelled P2 (p*_oriC−Br3_*) primer was used together with P1 (p*_oriC−Bf1_*) primer to amplify a 283-bp fragment of the *oriC* region for DNase I footprinting and sequencing reactions (see (*c*)). (*b*) EMSA. A ^32^P-labelled, 981-bp *oriC* fragment (PCR-amplified with p*_oriC−Bf1_* and p*_oriC−Br4_* primers, table 2) was incubated in Marians' buffer in the presence of a non-specific competitor with increasing amounts of AdpA protein, and the nucleoprotein complexes were analysed on a 4 per cent polyacrylamide gel. (*c*) DNase I footprinting of wild-type and *Δ*A1A2 *oriC* fragments. A ^32^P-labelled, 283-bp DNA fragment (see (*a*)) was incubated in Marians' buffer with increasing amounts of AdpA protein and then subjected to DNase I digestion. Lanes: A, C, G and T represent sequencing reactions for the WT and *Δ*A1A2 *oriC* fragments, respectively. Sequences above the gel schematically represent mutations introduced within *oriC* region (blue, nucleotides to be mutated; underlined, introduced nucleotides). Radiolabelled P2 (p*_oriC−Br3_*) primer was used to perform sequencing reactions. (*d*) *In vivo* identification of the AdpA–*oriC* complex using immunoprecipitation. Anti-AdpA polyclonal antibodies [24] were used to immunoprecipitate AdpA–DNA complexes cross-linked with formaldehyde. PCR was carried out with the primers, p*_oriC−Bf1_* and p*_oriC−Br3_* (see (*a*) and electronic supplementary material, table S2), flanking the putative AdpA-binding sites. Lanes: S, sample (immunoprecipitated DNA); plus symbols (+), input (not immunoprecipitated) DNA (positive control); strains: 1, strain containing the *adpA* gene under the control of the inducible promoter, M851 *p_tipA_adpA*; 2, *adpA* deletion mutant M851+pIJ6902-*hyg*.

As in other organisms, replication of the *Streptomyces* chromosome begins with the formation of an initiation complex at the origin region. Compared with other bacterial origins, *Streptomyces oriC* is longer and more complex; it contains 19 DnaA boxes, organized in two groups separated by a spacer (see figure 1*a* and [25]). Mutational analysis of individual DnaA boxes showed that replication depends on all DnaA boxes as well as on the spacer [25].

Here, we show for the first time that AdpA, the most extensively studied *Streptomyces* master regulator of aerial mycelium formation, also inhibits the initiation of chromosome replication. We demonstrate that AdpA specifically binds the *oriC* region and decreases DnaA protein access to the replication origin. Moreover, we provide evidence suggesting that AdpA and DnaA proteins compete for *oriC* binding in an ATP-dependent manner.

## 3. Results

### 3.1. AdpA interacts *in vivo* with the origin of replication in *Streptomyces coelicolor*

In order to identify replication origin binding protein(s) that might control initiation in *S. coelicolor*, we applied streptavidin affinity chromatography using biotinylated *oriC* as bait (see electronic supplementary material, materials and methods for detailed information). A 42.8 kDa protein was fished out from a cell extract prepared from a sample of a 44-hour-old *S. coelicolor* M145 culture (which had already formed aerial hyphae) growing on solid minimal medium supplemented with 1 per cent mannitol, but not from the extract of younger cultures. The protein was identified by mass spectroscopy (28% protein sequence coverage) as AdpA—a master transcriptional regulator of aerial mycelium formation [22,26,27]. In order to verify that AdpA was able to bind *oriC in vivo*, we performed formaldehyde cross-linking of proteins to DNA in intact cells followed by selective immunoprecipitation of protein–DNA complexes with anti-AdpA antibodies, as described previously [24]. As noted in the previous publication, we encountered difficulties with the cross-linking reaction in cultures grown on a solid medium, and therefore used the *S. coelicolor* M851 *p_tipA_adpA* strain, which is capable of producing AdpA in liquid culture upon induction with thiostrepton [24]. AdpA–*oriC* complexes were indeed detected after induction of this strain (set #1, lane S, figure 1*d*)—intensity of this band was comparable with the positive control (set #1, lane +)—whereas no signal was observed in the *adpA* deletion strain M851+pIJ6902-*hyg* (set #2, lane S), which served as negative control. These results indicate capability of forming AdpA–*oriC* complexes *in vivo*.

In summary, AdpA, a well-known transcription factor involved in the formation of aerial hyphae, binds the *oriC* region *in vitro* and *in vivo*. This suggests that AdpA might play a role in addition to that of transcriptional regulator, namely regulating the initiation of chromosome replication.

### 3.2. AdpA specifically binds the 5′-end of the *oriC* region

**Figure 2.**
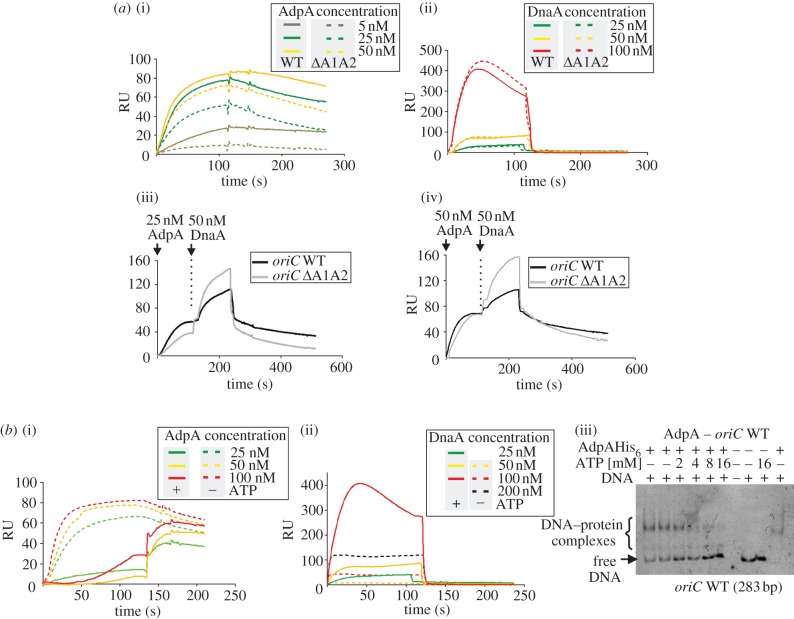
AdpA and DnaA proteins compete for *oriC-*binding in an ATP-dependent manner. (*a*) Binding of AdpA reduces accessibility of the *oriC* to DnaA. SPR analysis: comparison of AdpA and DnaA protein interactions with wild-type and *Δ*A1A2 *oriC* fragments. Sensograms were obtained by binding AdpA (i), DnaA (ii), or both proteins (co-injection, (iii) and (iv)) to biotinylated wild-type and *Δ*A1A2 *oriC* fragments (283-bp, amplified with biotinylated p*_b−Scoric_* and p*_oriC−Br3_* primers) immobilized on a streptavidin-coated chip in the BIAcore apparatus. Proteins were injected in HBS200 buffer supplemented with 3mM ATP (for DnaA) or without this nucleotide (for AdpA). (*b*) Presence of ATP reduces the affinity of AdpA for the *oriC* region. (i)–(ii) SPR: comparison of AdpA and DnaA protein interactions with the *oriC* fragment in the presence and absence of ATP. Sensograms were obtained by incubating AdpA (i) and DnaA (ii) proteins with biotinylated wild-type *oriC* fragment (283-bp, amplified with biotinylated p*_b−Scoric_* and p*_oriC−Br3_* primers) immobilized on a streptavidin-coated chip of the BIAcore apparatus. The concentration of ATP was 3 mM. (iii) Cross-linking of AdpA–*oriC* complexes formed in the presence or absence of ATP. A 283-bp DNA fragment (100 ng) was incubated with AdpA protein (100 nM) in the absence or presence of increasing amounts of ATP, and then nucleoprotein complexes were cross-linked with glutaraldehyde (final concentration: 0.5 mM). After electrophoresis (5% polyacrylamide), the gel was stained with ethidium bromide and analysed.

An *in silico* search allowed identification of three putative AdpA-binding sequences (named A1–A3 boxes) at the conventional 5′-end of *oriC*. Their sequences differed from the consensus 5′-TGGCSNGWWY-3′ [22] by one nucleotide, and two A boxes (A1 and A2) partially overlapped (figure 1*a*,*c*). Electrophoretic mobility shift assay (EMSA) and surface plasmon resonance (SPR) demonstrated that AdpAHis6 specifically bound the *oriC* fragment (283-bp) containing the *in silico*-predicted A-boxes in a concentration-dependent manner (figures 1*b* and 2*a*), but not the remaining part of *oriC* (data not shown). Additionally, EMSA showed the presence of one nucleoprotein complex at a low AdpAHis6 concentration, whereas increasing protein concentrations resulted in the formation of a ladder of higher-molecular-mass complexes (figure 1*b*). Moreover, the SPR analysis (figure 2*a*) revealed that dissociation of AdpAHis6 was slow, suggesting that the AdpA–*oriC* complexes are fairly stable. DNase I footprinting confirmed binding of the AdpA to the two *in silico*-predicted overlapping A boxes (figure 1*c*).

Taken together, our results demonstrate that AdpA specifically binds A boxes within the 5′ end of the *S. coelicolor oriC* region and forms a large, stable nucleoprotein complex.

### 3.3. AdpA and DnaA proteins compete for *oriC*-binding in an ATP-dependent manner

The two overlapping A boxes (A1 and A2) are directly flanked by weak DnaA boxes (1 and 2) containing two mismatches from the consensus (figure 1*a*). Our previous mutational analysis of individual DnaA boxes showed that replication of minichromosomes depends on all DnaA boxes, including those that are located next to the A boxes [28]. Because AdpA binding led to extended DNA protection that partially overlapped the functional DnaA boxes (DnaA boxes 1 and 2), we expected that accessibility of the weak DnaA boxes to DnaA protein would be limited in the presence of AdpA protein. To demonstrate that AdpA modulates DnaA accessibility to *oriC*, we mutated the two overlapping A boxes, A1 and A2, in such a way that the sequences of the adjacent DnaA boxes (1 and 2) and the distance between them were not altered. A subsequent DNase I footprinting experiment clearly showed that the mutated region (*oriC* *Δ*A1A2) was not protected by AdpA (figure 1*c*). SPR analyses revealed that AdpA protein exhibited lower affinity towards *oriC* *Δ*A1A2 than towards wild-type *oriC* (*oriC* WT), but the mutation did not alter the affinity of DnaA protein (figure 2*a*(i,ii)). Mutation of the A1A2 boxes reduced binding of AdpA to the *oriC* fragment and destabilized the nucleoprotein complex formed by increasing the dissociation of AdpA from *oriC* *Δ*A1A2 (figure 2*a*(i)). Co-injection experiments showed that binding of AdpA to the analysed DNA fragments considerably reduced binding of DnaA protein to *oriC* WT compared with *oriC* *Δ*A1A2 (see association curves, figure 2*a*(iii,iv)). This finding demonstrates that AdpA competes with DnaA for the *oriC* fragment containing A boxes, considerably reducing the accessibility of *oriC* to DnaA.

It should be noted that *S. coelicolor* DnaA, like that of *Mycobacterium tuberculosis* [29], binds DnaA box(es), particularly weak ones, more efficiently in the presence of ATP (figure 2*b*(ii)). Interestingly, we found that the affinity of AdpA protein for A boxes was substantially reduced in the presence of ATP. As shown in SPR analyses (figure 2*b*(i)), ATP at the same concentration used for DnaA-binding experiments nearly completely abolished binding of AdpA to the AdpA boxes. To confirm this intriguing observation, we cross-linked AdpA and DNA in the presence of increasing amounts of ATP (figure 2*b*(iii)). In this assay, ATP prevented the formation of AdpA–DNA complexes in a concentration-dependent manner, an outcome consistent with the results obtained by SPR analysis. Interestingly, a similar effect was also observed in the presence of ADP (see electronic supplementary material, figure S1b), which suggests that the energy status (ATP/ADP) of the cell does not exert influence on binding of this protein to DNA.

In summary, our results show that AdpA and DnaA proteins compete for *oriC*-binding in an ATP-dependent complementary manner, such that the presence of ATP favours DnaA binding, and the absence of ATP favours AdpA binding.

### 3.4. Mutation of an AdpA-binding motif within *oriC* influences colony maturation and replication rate

**Figure 3.**
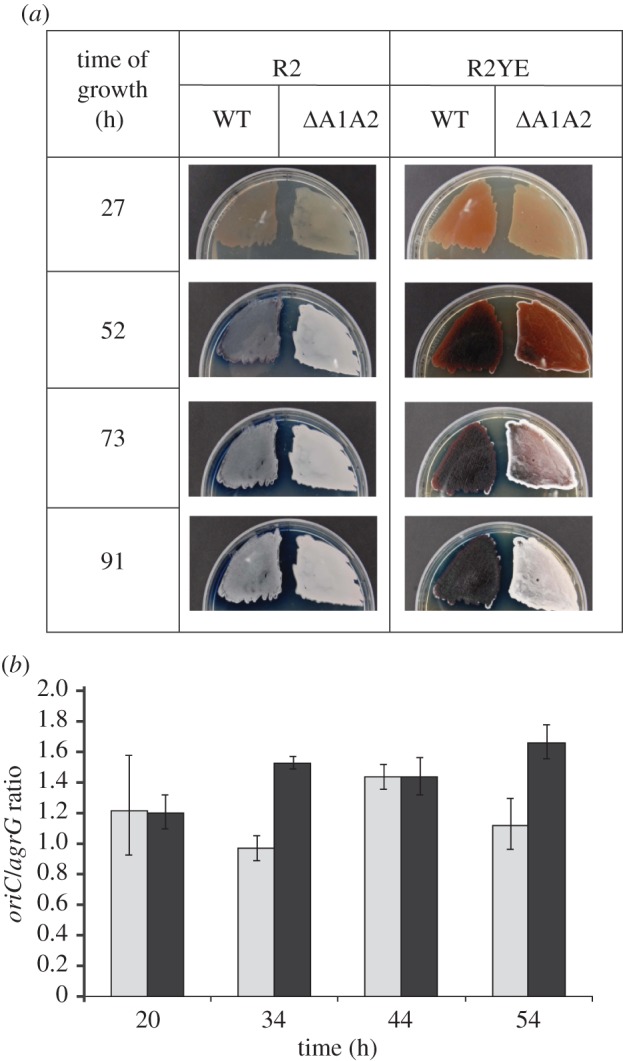
Mutation of AdpA-binding sites within the *oriC* region results in earlier maturation of aerial hyphae and more frequent replication. (*a*) Growth of wild-type and *Δ*A1A2 mutant strains of *S. coelicolor* on R2 and R2YE media. (*b*) Quantitative PCR analysis of frequency of initiation in wild-type and *Δ*A1A2 mutant strains of *S. coelicolor* on R2 medium. Real-time qPCR analyses were performed as described in §5 (see electronic supplementary material to quantify the ratio of *oriC* to *argG*, which reflects the frequency of initiation). Grey bars, M145; black bars, *Δ*A1A2.

Because AdpA, acting as a transcription factor, binds promoters of many genes that are important for the formation of aerial hyphae [21,22], we expected that binding of AdpA to the *oriC* region might also have a specific function during differentiation of *Streptomyces*. To investigate the biological role of AdpA–*oriC* complex formation, we constructed the *S. coelicolor* *Δ*A1A2 strain, in which a mutation of the overlapping A1 and A2 boxes (the same mutation as described earlier) was introduced within the native *oriC* locus (for details see electronic supplementary material). The mutant formed aerial hyphae (visible as a white confluent fluffy layer) substantially earlier than the wild-type strain, particularly on R2YE medium (figure 3*a*): aerial hyphae were already noticeable within 52 h, whereas an aerial hyphal layer was poorly visible even at 91 h in the wild-type strain (figure 3*a*). The expression profile of the *adpA* gene was not altered in the mutant strain compared with the wild-type strain (data not shown), ruling out unexpected changes in *adpA* expression as an explanation for the accelerated development.

These results suggested that AdpA might be involved in the regulation of chromosome replication—specifically, by acting as an inhibitor of chromosome replication, particularly affecting aerial growth. To examine this hypothesis, we quantified the abundance of *oriC* region copies and chromosome terminus (*ter*) copies on chromosomal DNA isolated from wild-type and mutant strains using a real-time polymerase chain reaction (RT-PCR) analysis (figure 3*b*). In this analysis, which used primers targeting sites near *oriC* (*gyrB* gene, centrally located on the linear chromosome) and *argG*, close to one end of the chromosome, the *oriC*-to-*argG* ratio reflects the frequency of replication initiation. The ratio was normalized internally using DNA isolated from spores, which are replication-inactive. At early stages of growth when AdpA was expressed at low level (20 h) [24], the *oriC*/*argG* ratio for the mutant strain was similar to that of the wild-type strain. However, as aerial hyphae became visible and AdpA was synthesized at higher levels (34 h; the highest AdpA levels were observed at around 36 h and remained high within subsequent hours [24]), the ratio was about 50 per cent higher for the mutant strain than for the wild-type strain, indicating more frequent initiation in the mutant compared with the wild-type, particularly at 54 h, when the wild-type ratio subsided to unity, but the mutant ratio remained high. The fluctuations of the ratio in the wild-type and the mutant (the same values at 44 h; figure 3*a*) may be results of the decrease in AdpA concentration between 36 h and 48 h reported previously [24]. Thus, these results support the idea that AdpA acts as a repressor of replication initiation prior to the emergence of aerial hyphae by binding to *oriC-*linked AdpA-binding sequences, and that mutation of these sequences increased the accessibility of *oriC* to DnaA, resulting in earlier replication in substrate mycelium compartments capable of giving rise to aerial branches. Interestingly, in the wild-type strain containing an additional copy of *adpA* gene under the inducible promoter, p***_tipA_***, we observed slowed aerial mycelium formation (data not shown), which supports the idea mentioned earlier. The high ratio observed in the 54 h time point with the mutant might suggest that AdpA also affects replication at the time of sporulation, but further analysis of the mutant will be needed to determine whether its development at this stage is normal (for example, the premature growth of aerial hyphae might be followed by another round of aerial growth as the vegetative mycelium continues to accumulate biomass).

## 4. Discussion

In *Streptomyces*, chromosome replication takes place along much of the vegetative and aerial hyphae, although replication is particularly intensive in the rapidly extending apical compartments of the aerial mycelium, giving dozens of chromosomes that are then segregated into unigenomic spores. On the other hand, replication levels are much reduced in those compartments of the substrate mycelium that do not contain a tip, such as those that will eventually give rise to an aerial branch. Thus, both in the non-growing compartments and at the time of sporulation, a mechanism (or mechanisms) that prevents subsequent rounds of replication needs to be switched on.

We present data here showing that AdpA—a key regulator of aerial mycelium formation—controls chromosome replication at the initiation step by binding to the *oriC* region. Using affinity chromatography with *S. coelicolor oriC* as bait, we fished out AdpA from cell extracts of cultures undergoing aerial mycelium development (AdpA reaches a maximum concentration at an early stage of aerial mycelium formation [24]). Different *in vivo* and *in vitro* approaches revealed that the AdpA protein bound specifically to two *in silico-*predicted A boxes (A1A2) located in the 5′-end of the *oriC* region (figure 1). Moreover, the A boxes partially overlap with DnaA-binding sites that are indispensable [28] for the initiation of replication, suggesting possible competition between transcription factor and initiator protein for *oriC* binding. A mutation of the A1A2 boxes that lowered affinity of AdpA protein for *oriC* considerably increased accessibility of the replication origin to DnaA protein (figure 2*a*(iii,iv)). These results imply that AdpA is responsible for the inhibition of DnaA binding and chromosome replication. However, the competition between AdpA and DnaA proteins is not straightforward. ATP seems to be a key regulator of the DNA-binding activity of both proteins. It is well known that ATP strengthens binding of DnaA protein to DnaA boxes, particularly weak ones (figure 2*b*(ii)), and is required for DNA unwinding [30]. Unexpectedly, we found that ATP inhibits AdpA binding to *oriC*: in the presence of ATP, the affinity of AdpA for A1A2 boxes was considerably diminished (figure 2*b*(i)). However, the mechanism responsible for this phenomenon is not yet known because AdpA does not contain a recognizable ATP-binding motif; preliminary results suggest that ATP does not affect binding affinity of the truncated form of AdpA encompassing only the DNA-binding domain (see electronic supplementary material, figure S1a).

The *in vivo* analysis of replication frequency (*oriC* to *argG* ratio determined by qRT-PCR) in the *S. coelicolor* *Δ*A1A2 strain (with mutated A1A2 boxes) and wild-type strain are in line with *in vitro* studies suggesting that AdpA blocks initiation of replication: mutations of AdpA-binding sites within the *oriC* region increased initiation frequency by about 50 to 60 per cent compared with the wild-type strain, at a time point when aerial hyphae were emerging and at a later time point when, in the wild-type, sporulation was in progress. The observed increase in initiation frequency was not dramatic, but it should be noted that replication in multigenomic hyphae is an asynchronous process [12]. Additionally, *S. coelicolor* differentiates only on solid media, and aerial hyphae, which constitute only approximately 10 per cent of the total biomass, cannot be separated from the non-differentiating vegetative mycelium. Therefore, only changes in replication initiation frequency of at least a few-fold can actually be detected in aerial hyphae.

Our findings suggest a novel role for AdpA—a transcription factor thus far known as a master regulator of genes involved in aerial mycelium development—and allow us to propose a model for ATP-dependent regulation of chromosome replication during formation of aerial hyphae. Our model is based on the assumption that in *Streptomyces*, as in other organisms, both the level of the active form of DnaA (DnaA–ATP) [31] and the level of ATP [32] fluctuate during the cell cycle. *Streptomyces* DnaA, similarly to *E. coli* DnaA [5] and eukaryotic ORC (origin recognition complex) [33], is probably associated with *oriC* (via high-affinity DnaA box(es), e.g. box number 6, figure 1*a* [25]) throughout most of the cell cycle, independent of the nucleotide-bound state of DnaA. We assume that in the wild-type, older substrate hyphal compartments are subject to nutrient limitation, and therefore probably to ATP depletion. Presumably, at the same time (approx. 36 h), AdpA reaches its highest levels [24], which presages the emergence of aerial branches. We suppose that owing to those two mentioned factors (low ATP level and high AdpA concentration), DnaA is not able to efficiently bind to the *oriC* region and initiate replication. This would persist until ATP levels increase (partially as stored nutrients are mobilized), triggering dissociation of AdpA from *oriC*, and permitting binding and activation of DnaA, and the initiation of replication associated with emergence of an aerial branch. The accelerated development of aerial hyphae in the *S. coelicolor* *Δ*A1A2 strain, in which DnaA has easier access to the *oriC* region, is consistent with this model. It is also possible that AdpA plays a similar role later in development, in the cessation of replication initiation accompanying sporulation septation, but further analysis of the *Δ*A1A2 mutant phenotype will be needed to evaluate this possibility. Interestingly, all *Streptomyces* genomes sequenced so far possess genes encoding AdpA proteins that share high amino acid sequence similarity; moreover, AdpA boxes are present in *oriC* regions of certain *Streptomyces* strains (*S. griseus*, *Streptomyces lividans*, *Streptomyces avermitilis*; data not shown). Thus, we suppose that AdpA also acts as a regulator of chromosome replication in other *Streptomyces* species. At least one other mechanism may predominate at the time of sporulation septation, when chromosomes are condensed [34] and segregated by ParA and ParB [35] into prespore compartments, and the formation of large ParB complexes around the *oriC* region [36] may prevent re-initiation. Recently, it has been demonstrated that the *B. subtilis* ParB orthologue, Spo0J, is also involved in the regulation of replication initiation, but the mechanism of its action is different; Spo0J regulates the oligomeric state of Soj (ParA) to trigger its switch from an activator to an inhibitor of chromosome replication initiation [37].

Switching off replication by ‘arresting’ of the *oriC* region also occurs in other bacteria that undergo cellular differentiation. Thus far, two global regulators, Spo0A and CtrA, which block the initiation of replication in the spore-forming bacterium *B. subtilis* and in the dimorphic bacterium *Caulobacter crescentus*, respectively, have been identified. These regulators specifically bind *oriC* before formation of endospores in *B. subtilis* (Spo0A) [8] or in the flagellated daughter cell of *C. crescentus* (CtrA) [38]. However, in contrast to *Streptomyces*, they do not compete with DnaA for the *oriC* region in an ATP-dependent manner. Instead, their activity is modulated during the cell cycle by phosphorylation [39].

In summary, AdpA, a key transcription factor involved in regulating aerial hyphae formation, inhibits chromosome replication at the initiation step by binding the 5′-end of the *oriC* region. Mutation of AdpA-binding sequences in the *oriC* region leads to over-replication and earlier formation of aerial hyphae. Surprisingly, we showed here that, in contrast to DnaA, AdpA affinity for the *oriC* region was considerably diminished in the presence of ATP. To our knowledge, a mechanism for regulation of chromosome replication based on ATP-dependent competition between transcription factor (AdpA) and initiator protein (DnaA) is without precedent among both bacteria and eukaryotes. It should be noted in this context that earlier studies showed that ATP might serve as an intracellular effector to control the synthesis of secondary metabolites (antibiotics) [40,41].

## 5. Material and methods

### 5.1. Bacterial strain growth conditions, DNA manipulation and protein purification

The *E. coli* and *S. coelicolor* strains used are listed in the electronic supplementary material, table S1. Media, culture conditions and DNA manipulations were as described previously [24]. Total DNA was isolated from *S. coelicolor* mycelium grown on cellophane-covered R2 agar as described previously [24]. An *S. coelicolor* mutant (*S. coelicolor* *Δ*A1A2) carrying a mutation of the AdpA-binding site within the *oriC* region was constructed using a PCR-based targeting procedure and homologous recombination (see electronic supplementary material, materials and methods for detailed information). The fusion proteins AdpAHis_6_ and DnaAHis_6_ were purified using affinity chromatography (HIS-Select HF resin), as described previously by [24].

### 5.2. *In vivo* immunoprecipitation

The immunoprecipitation assay was performed as described previously [24,36]. Briefly, *S. coelicolor* strains (*adpA* deletion mutant and the strain containing the *adpA* gene under the inducible promoter, p***_tipA_***), cultivated for 24 h in 79 liquid media, were treated with formaldehyde to cross-link nucleoprotein complexes that were then immunoprecipitated with anti-AdpA antibodies [24,36]. Cells that were not subjected to cross-linking but otherwise treated in the same way as experimental samples served as negative controls. Immunoprecipitated DNA was PCR-amplified using primers flanking the AdpA-binding sites.

### 5.3. Real-time PCR analysis of the frequency of replication initiation

The ratio of the number of copies of the *oriC* region to the number of copies of the chromosome end, reflecting the frequency of replication initiation, was measured using a quantitative PCR method. The primers (designed with PrimerExpress v. 3.0) used for amplification of the *oriC* region (*gyrB* gene; pForGYRB, pRevGYRB) and chromosome terminus (*argG* gene; pForARG, pRevARG) are indicated in the electronic supplementary material, table S2. Quantitative PCR was performed on a StepOne Plus Real-Time PCR System (Applied Biosystems) using Real-Time 2× PCR Master MIX SYBR A kit (A&A Biotechnology) and the following parameters: 95°C for 10 min, followed by 40 two-step amplification cycles consisting of 15 s at 95°C (denaturation) and 1 min at 60°C (annealing and extension). Reaction mixtures (20 µl) contained chromosomal DNA (10 ng) and primers (8 pmol each). The specificity of the PCR products was verified by performing a melting curve analysis. A negative control (H_2_O instead of DNA) was included in all real-time PCR assays. Observed cycle thresholds (Ct) allowed determination of a calibrator-normalized relative quantity of *oriC*/*argG* (represented by *gyrB*-to-*argG* ratio) for each sample using StepOne v. 2.0 software (Applied Biosystems). To determine relative abundance of *oriC*, we used DNA isolated from spores in which replication does not take place (*oriC*/*argG* = 1) as a calibrator, and the *oriC* level was normalized using *argG* as internal standard. Each experiment was performed in triplicate.

### 5.4. Electrophoretic mobility shift assay

EMSAs were carried out as described previously [24]. Briefly, a ^32^P-labelled DNA fragment was incubated with increasing amounts of purified AdpAHis6 protein in the presence of the non-specific competitor poly(dI-dC)•(dI-dC) in 1× Marians' buffer (20 mM HEPES/KOH pH 8.0, 5 mM MgOAc, 1 mM EDTA, 4 mM dithiothreitol, 0.2% Triton X-100, 5 g l^−1^ BSA and 5% glycerol) for 30 min at room temperature. The nucleoprotein complexes were resolved on 4 or 5 per cent polyacrylamide gels at approximately 8°C in 0.25× TBE buffer at 5–10 V cm^−1^. Complexes were analysed with a Typhoon 8600 Variable Mode Imager and Image Quant software.

### 5.5. *In vitro* cross-linking of DNA–protein complexes

AdpAHis6 protein (final concentration, 100 nM) in 1× HBS200 buffer (10 mM HEPES pH 7.4, 10 mM MgOAc, 200 mM NaCl, 3.4 mM EDTA, 0.05% Tween 20) was incubated for 10 min at 25°C with the indicated concentration of ATP. DNA (100 ng in 1× HBS200 buffer) was then added to the reaction mixture and incubated for 30 min at 25°C. DNA–protein complexes were preserved in solution by adding the cross-linking agent glutaraldehyde (final concentration, 0.5 mM) at the last step of the procedure and incubating for 5 min at 25°C. After chilling on ice, the entire sample (20 µl) was applied to a 5 per cent polyacrylamide gel and electrophoresed in 0.25× TBE buffer at 5–10 V cm^−1^ at 8°C. After electrophoresis, the gel was stained with ethidium bromide (0.5 µg ml^−1^) and analysed with a Typhoon 8600 Variable Mode Imager and Image Quant software.

### 5.6. DNase I footprinting

A 283-bp PCR product encompassing the 5′ portion of the *oriC* region, obtained by amplification with the primers poriC-Bf1 and poriC-Br3, was used for DNase I footprinting experiments. The 5′-end-radiolabelled DNA fragments (approx. 10 fmol) were incubated with different amounts of AdpAHis6 protein in 1× Marians' buffer at 25°C for 30 min. Phosphate ions, which affect DNase I activity, were removed by dialysing the AdpAHis6 protein solution overnight against buffer DIAdpA (20 mM HEPES pH 8.0, 5 mM MgOAc, 1 mM EDTA pH 8.0) before use. After DNase I digestion [42], cleavage products were separated on an 8 per cent polyacrylamide-urea sequencing gel. The gel was analysed with a Typhoon 8600 Variable Mode Imager and Image Quant software.

### 5.7. Surface plasmon resonance analysis

For standard SPR analyses, a 283-bp fragment of *oriC* region was PCR-amplified with biotinylated pb-Scori primer and non-biotinylated poriC-Br3 primer, and then immobilized on the chip surface (Sensor Chip SA, GE Healthcare) in a BIAcore 3000 apparatus; approximately 100 response units (RUs) of DNA were immobilized. A non-AdpA box/non-DnaA box DNA fragment was used as a negative control. DNA loosely attached to the surface of the chip was removed with a 5 s pulse of 0.1 per cent SDS (15 µl min^−1^). Potential effects of mass transport on the kinetics of the protein–DNA interactions were excluded by performing measurements at different AdpAHis6 and DnaA protein concentrations (5–200 nM) and at a continuous flow rate (15 µl min^−1^) for 120 s. Measurements were performed in HBS200 buffer (10 mM HEPES pH 7.4, 10 mM MgOAc, 200 mM NaCl, 3.4 mM EDTA, 0.05% Tween 20) supplemented with 3 mM ATP or without this nucleotide; HBS200 buffer without ATP was used as a running buffer. At the end of each cycle, bound proteins were removed by washing with a 5 s pulse of 0.1 per cent SDS (15 µl min^−1^). The results were plotted as sensorgrams after subtraction of the background response signal obtained in a control experiment. The BIAevaluation v. 4.1 program (Pharmacia Biosensor AB) was used for data analysis.

## Supplementary Material

Supplementary figure

## Supplementary Material

List of strains and plasmids, PCR primers used, Supplementary materials and methods
