# Supplementary figures and images for: AdpA, key regulator for morphological differentiation regulates bacterial chromosome replication
Source: Open Biol. 2012 Jul;2(7):120097. doi: 10.1098/rsob.120097 (PMC3411110; doi:10.1098/rsob.120097)

(a)

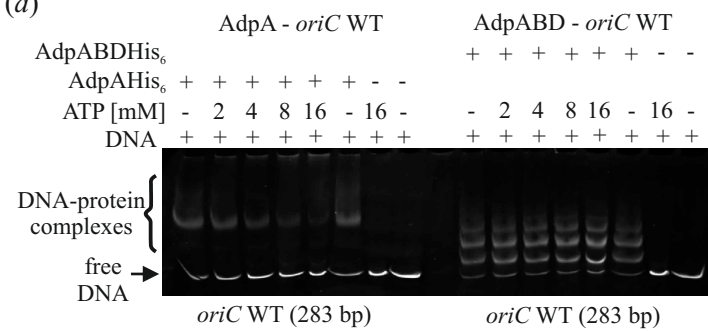

(b)

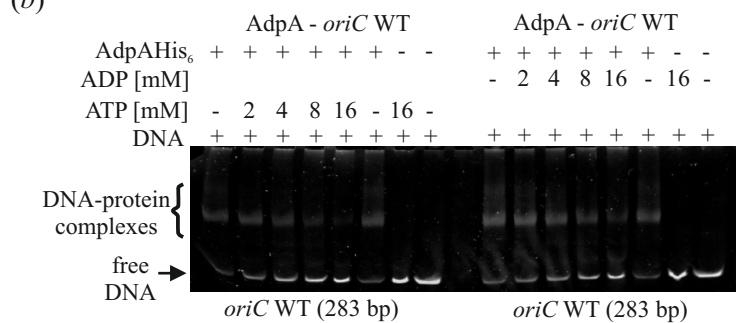

Supplement: Supplementary figure [file rsob120097-s1.pdf]
