# Supplementary material for: AdpA, key regulator for morphological differentiation regulates bacterial chromosome replication
Source: Open Biol. 2012 Jul;2(7):120097. doi: 10.1098/rsob.120097 (PMC3411110; doi:10.1098/rsob.120097)
Supplement: List of strains and plasmids, PCR primers used, Supplementary materials and methods [file rsob120097-s2.docx]

**Supporting information**

**Supplementary materials and methods:**

***Strain construction – mutation of AdpA sequences***

A PCR-based targeting procedure was employed to introduce a mutation of the AdpA-binding site into the *oriC* of *Streptomyces coelicolor* to yield the strain, *S. coelicolor*ΔA1A2. In the ﬁrst round, an apramycin-resistance cassette was amplified from the pIJ773 plasmid (DNA template) (Gust et al, 2003) using the primers pAdpA_mut_fw and pAdpA_mut_rv, which introduced *Nde*I restriction sites at both ends of the cassette. The resulting PCR product was inserted within the AdpA-binding site in the *oriC* region of the StH18 cosmid and introduced into arabinose-induced *E. coli* BW25113/pIJ790 strain. Cosmid DNA was isolated from apramycin-resistant colonies and verified by restriction with *BamH*I. A positive cosmid clone was subsequently digested with *Nde*I and ligated to restore a natural-length *oriC* region through precise excision of the antibiotic cassette, leaving an *Nde*I site in place of the AdpA-binding site. The presence of the *Nde*I site was confirmed by *Nde*I digestion of purified PCR product obtained by amplification with the primers oriC-Bf1 and oriC-Br3 using the cosmid as a template. The resulting cosmid, which lacked *oriT* (*ori*gin of *t*ransfer), was subjected to a second round of the PCR-based targeting procedure to allow transfer of the cosmid from *E. coli* to *S. coelicolor*. This was accomplished by targeting the *bla* gene in the cosmid backbone with the apramycin resistance gene containing *oriT* (PCR product amplified with bla_p1 and bla_p2 primers using pIJ773 plasmid DNA as a template) using a procedure similar to that described previously (Jakimowicz et al, 2007). The resulting cosmid (StH18ΔA1A2) was then transferred to ET12567/pUZ8002 strain, from which it was mobilized into *S. coelicolor* M145 by conjugation. Kanamycin-resistant colonies indicative of a single-crossover event were selected. Then, transformants were selected for loss of apramycin and kanamycin resistance. Chromosomes isolated from selected clones were verified by restriction digestion of PCR products with *Nde*I, as described for verification of the presence of the *Nde*I site.

***Affinity chromatography***

The *oriC* fragment (981 bp) was amplified by PCR using the 5’-biotin-labelled forward primer pb-Scori and poriC-Br4 (Table S2). The resulting biotinylated *oriC* (10 pmol) was immobilized on Streptavidin Magnetic Dynabeads (Dynabeads kilobase BINDER Kit, Dynal Biotech). *S. coelicolor* lysates were prepared from cultures grown on cellophane discs on solid minimal medium supplemented with 1% mannitol. At the indicated time points, mycelia were scraped off the cellophane surface and immediately ground in liquid nitrogen and stored at ‑70°C. Before use, homogenized cultures were thawed on ice and resuspended in chilled phosphate buffered saline (PBS: 0.8% NaCl, 0.02% KCl, 0.144% Na_2_HPO_4_, 0.024% KH_2_PO_4_) supplemented with 1 mM EDTA and protease inhibitors (Complete Protease Inhibitor Cocktail Tablets, Roche). Samples were then sonicated on ice (5 x 30-s pulses, with 30-s intervals between pulses) and centrifuged for 10 min at 13,000 x g at 4°C. Protein concentration in supernatants was determined using the Bradford assay (Bradford, 1976). For “fishing” experiments, 6 mg of total protein extract (final volume, 14 ml) was incubated with 10 pmol of DNA immobilized on Dynabeads with constant gentle mixing for 1 h at 25°C. Dynabeads were subsequently washed and eluted with PBS buffer supplemented with increasing NaCl concentrations. Proteins in eluates were resolved by SDS-PAGE on 10% gels, and gels were stained with Coomassie brilliant blue. Visible protein bands were excised from the gel and analyzed by mass spectrometry.

***Construction of the AdpA truncated form (AdpA binding domain)***

The *adpA* gene fragment encoding DNA binding domain of AdpA protein (AdpABD) was PCR-amplified using IIadpA-BF and adpAXRV oligonucleotides and cloned into pET-21a(+) vector and then overexpressed as a C-terminal His-tagged protein in *E. coli* BL21 strain (Wolański et al, 2011). The purified AdpA***_c_***His_6_ protein was more than 95% pure (as judged by SDS-PAGE analysis, data not shown). Obtained clones were analyzed by DNA sequencing to check their fidelity.

(Oligonucleotides sequences: IIadpA-BF, GGATCCCAGGAGCGCTACCTCGACAGGTC, *BamH*I site underlined;

adpAXRV, CTCGAGCGCGCTGCGCTGGCCCGGG, *Xho*I site underlined)

**References**

Bradford M M. 1976 A rapid and sensitive method for the quantitation of microgram quantities of protein utilizing the principle of protein-dye binding. *Anal. Biochem.* **72**, 248-254.

Gust B, Challis G L, Fowler K, Kieser T, Chater K F. 2003 PCR-targeted *Streptomyces* gene replacement identifies a protein domain needed for biosynthesis of the sesquiterpene soil odor geosmin. *Proc. Natl Acad. Sci. U S A* **100**, 1541-1546. (doi:10.1073/pnas.0337542100)

Jakimowicz D, Zydek P, Kois A, Zakrzewska-Czerwinska J, Chater K F. 2007 Alignment of multiple chromosomes along helical ParA scaffolding in sporulating *Streptomyces* hyphae. *Mol. Microbiol.* **65**, 625-641. (doi:10.1111/j.1365-2958.2007.05815.x)

Wolański M, Donczew R, Kois-Ostrowska A, Masiewicz P, Jakimowicz D, Zakrzewska-Czerwinska J. 2011 The Level of AdpA directly affects expression of developmental genes in *Streptomyces coelicolor*. *J. Bacteriol.* **193**, 6358-6365. (doi:10.1128/JB.05734-11)

**Supplementary figure legend:**

**Fig. S1. Influence of ATP and ADP on the DNA binding activity of AdpA and its truncated form, AdpABD.**

**(*a*). Cross-linking of AdpA and AdpABD-*oriC* complexes formed in the presence or absence of ATP**. A 283-bp DNA fragment (100 ng) was incubated with AdpA or AdpABD (AdpA binding domain) protein (100 nM) in the absence or presence of increasing amounts of ATP, and then nucleoprotein complexes were cross-linked with glutaraldehyde (final concentration, 0.5 mM). After electrophoresis (5% polyacrylamide), the gel was stained with ethidium bromide and analyzed.

**(*b*). Cross-linking of AdpA-*oriC* complexes formed in the presence orabsence of ATP or ADP**. A 283-bp DNA fragment (100 ng) was incubated with AdpA protein (100 nM) in the absence or presence of increasing amounts of ATP or ADP, and then nucleoprotein complexes were cross-linked with glutaraldehyde (final concentration, 0.5 mM). After electrophoresis (5% polyacrylamide), the gel was stained with ethidium bromide and analyzed.

**Table S1. List of strains and plasmids**

| **Strain** | **Relevant genotype** | **Source** |
| --- | --- | --- |
| *E. coli* |  |  |
| DH5α | *supE44ΔlacU169(φ80lacZΔM15)hsdR17 recA1 endA1 gyrA96 thi-1 relA1* | Lab stock |
| BW25113/pIJ790 | K12 derivative: *ΔaraBAD, ΔrhaBAD λ-Red(gam,bet,exo), cat, araC, rep101^ts^* | (Gust et al,2003) |
| ET12567/pUZ8002 | *dam-*13::Tn*9*, dcm cat tet *hsd* *zjj-201::Tn10/tra neo* RP4 | (Kieser et al, 2000) |
| *S. coelicolor* |  |  |
| M145 | SCP1^-^, SCP2^-^ | (Kieser et al, 2000) |
| M851 | M145 derivative: *ΔadpA* | (Takano et al, 2003) |
| M851+pIJ6902 *hyg* | M851+pIJ6902-*hyg* | (Wolanski et al, 2011) |
| *S. coelicolor* M851 *p_tipA_adpA* | M851+pIJ6902/2528-*hyg* | (Wolanski et al, 2011) |
| *S. coelicolor*ΔA1A2 | M145 derivative: *ΔA1A2* | This study |
| **Plasmid** | **Relevant genotype** | **Source** |
| pIJ6902/2528-*hyg* | pIJ6902-*hyg* containing *adpA****_c_*** under the control of the thiostrepton-inducible *PtipA* promoter | (Wolanski et al, 2011) |
| pIJ6902-*hyg* | pIJ6902 derivative containing Hyg^R^ instead of Apra^R^ | (Wolanski et al, 2011) |

Gust B, Challis G L, Fowler K, Kieser T, Chater K F. 2003 PCR-targeted *Streptomyces* gene replacement identifies a protein domain needed for biosynthesis of the sesquiterpene soil odor geosmin. *Proc. Natl Acad. Sci. U SA* **100**, 1541-1546. (doi: 10.1073/pnas.0337542100)

Kieser T, Bibb M J, Buttner M J, Chater K F, Hopwood D A. 2000 *Practical Streptomyces genetics*. Norwich, England: John Innes Foundation.

Takano E, Tao M, Long F, Bibb M J, Wang L, Li W, Buttner M J, Bibb M J, Deng Z X, Chater K F. 2003 A rare leucine codon in *adpA* is implicated in the morphological defect of *bldA* mutants of *S. coelicolor*. *Mol. Microbiol.* **50**, 475-486.

Wolanski M, Donczew R, Kois-Ostrowska A, Masiewicz P, Jakimowicz D, Zakrzewska-Czerwińska J. 2011 The level of AdpA directly affects expression of developmental genes in *Streptomyces coelicolor. J. Bacteriol****.*** , **193**, 6358-6365. (doi:10.1128/JB.05734-11)

**S2 Table PCR primers used**

| **Primer name** | **Primer sequence (5’-3’)** | **Purpose** |
| --- | --- | --- |
| ForARG | CGTTCAAGGGCAACGACAT | Amplification of end region of chromosome - qPCR assay |
| RevARG | TAGATCCTCAGCTGCGGGTT | Amplification of end region of chromosome - qPCR assay |
| ForGYRB | GGCAACACCGAGGTGAAGA | Amplification of *oriC* region of chromosome - qPCR assay |
| RevGYRB | AGCCAGTCCGTCAGGTGCT | Amplification of *oriC* region of chromosome - qPCR assay |
| oriC-Bf1 | Ccaaccgcatcaagaacggctgac | Amplification of *oriC* region - EMSA assay and immunoprecipitation |
| oriC-Br3 | cttgcctgtggacagGATCGGG | Amplification of *oriC* region - EMSA assay and immunoprecipitation |
| oriC-Br4 | gtcgcgttccacccggatcttcac | Amplification of *oriC* region – affinity chromatography assay |
| b-Scori | ccaaccgcatcaagaacggctgac | Amplification of *oriC* region (5’ biotinylated primer) |
